# Parallel evolution of the elite neutralizer phenotype in divergent HIV-1 clades

**DOI:** 10.1128/jvi.00433-25

**Published:** 2025-08-21

**Authors:** Kathryn A. Mesa, Sophia W. Li, Jennie M. Hutchinson, Sara O'Rourke, David L. Alexander, Bin Yu, Xiaoying Shen, Terri Wrin, Christos J. Petropoulos, Phillip W. Berman, Grant H. Pogson

**Affiliations:** 1 Department of Biomolecular Engineering, Baskin School of Engineering, University of California Santa Cruz 8787, Santa Cruz, California, USA; 2 TrueBinding Inc, Foster City, California, USA; 3 Color Health Inc 716785 , Burlingame, California, USA; 4 Coherus BioScience, Camarillo, California, USA; 5 The Duke Human Vaccine Institute, Duke University 3065, Durham, North Carolina, USA; 6 Monogram Biosciences 53555 , South San Francisco, California, USA; 7 Department of Ecology & Evolutionary Biology, University of California Santa Cruz 8787, Santa Cruz, California, USA; Icahn School of Medicine at Mount Sinai, New York, New York, USA

**Keywords:** HIV-1, evolutionary biology

## Abstract

The development of an effective vaccine against HIV-1 requires understanding how broadly neutralizing antibodies (bNAbs) evolve in natural viral infections. Here, we recovered 152 envelope sequences from two elite neutralizers (ENs) and five viral controllers and determined the neutralization sensitivity (IC_50_) of each envelope glycoprotein (Env) to broadly neutralizing monoclonal antibodies (bN-mAbs). For the combined EN/controller data set, we observed that the median IC_50_ value for a CD4-binding site (CD4bs) bN-mAb (VRC01) was significantly lower for viruses lacking an N465 glycan. For a clade AE EN viral population, Env mutations implicating a VRC01-like antibody occurred in concert with positive selection at N465. Using rabbit immunizations, N465 was found to shield immunogenic VRC01 contacts in the V5/β24 domain. In the clade B EN viral population, positive selection was observed at N332, a CD4bs epitope, and a 2G12-like glycan-dependent epitope (N295-N339-N392). In rabbit immunizations, a recombinant antigen derived from the basal virus showed a greater median positivity response to cladal consensus peptides overlapping N332 than from a later virus. However, the latter virus, which displayed an N49 glycan, elicited a greater median response to consensus peptides overlapping a VRC01 contact in the C1 domain. Rarely observed C1 glycans (N49, N97) evolved in both EN viral populations. Signals of positive selection, while largely absent for glycan polymorphism present in the controllers, were detected at glycans shielding bNAb epitope contacts in both EN viral populations. Positive selection of shielding glycans suggested constraints on the escape pathways of glycan-dependent antibodies imposed by bNAb responses.

**IMPORTANCE:**

In a small subset of HIV-1-infected individuals, natural viral evolution leads to the appearance of antibodies capable of neutralizing a broad assemblage of viruses from divergent clades (bNAbs). In this study, we determined that two viral populations with divergent infections exhibited commonalities in glycan evolution that accompanied the acquisition of exceptional serum neutralization breadth. Positive selection of glycans shielding immunogenic bNAb epitope contacts suggested conflicts for escape from glycan-dependent antibodies, and rare C1 glycan introductions highlighted the immunogenicity of a region overlapping a VRC01 epitope contact. Conflicts for glycan loss could lead to their persistence in a viral population. However, a heightening of a regional cross-reactive immune response concomitant with extraordinary glycosylation pointed to evolution of specific sequence for expanding antibody neutralization breadth. These results suggest that antigens displaying immunogenic bNAb epitopes in combination with rare glycosylation might help realize the production of an effective vaccine.

## INTRODUCTION

The primary goal of HIV-1 vaccine development remains the induction of antibodies capable of neutralizing a broad range of heterologous viruses (bNAbs). As such antibodies have been isolated from rare HIV-1-infected individuals termed elite neutralizers (ENs) (1, 2), it is feasible that their induction is also achievable through vaccination. The ultimate success of this endeavor depends on understanding the underlying mechanisms driving natural viral evolution that culminates in the appearance of bNAbs in diverse infections (3–5).

Antibodies with breadth can develop after repeated cycles of immune escape where autologous neutralizing antibodies (Nabs) select for resistant viral variants (6–9) that drive increases in Nab cross-reactivity (10). Expansions in cross-reactivity might occur by viral escape variants that engage antibody sub-lineages showing greater tolerance of variation, or by steering antibody lineages with overlapping epitopes toward increasingly higher affinity via cooperative pathways (11–13). The latter mechanism (as described for the evolution of a CD4-binding site (CD4bs) bNAb) showed how viral escape driven by two overlapping B-cell lineages promoted bNAb maturation (9, 12, 14, 15). However, another source of immune selection known to drive bNAb development is glycan-dependent Nabs (5, 16).

The glycans that densely shield the surface of the envelope glycoprotein (Env) from the immune system (7) also form the epitopes of Nabs (2, 17), rendering them susceptible to removal by antibody-mediated selection. Glycan loss for viral escape from autologous Nabs can expose underlying protein (18), increase oligomannose processing (19, 20), or disrupt viral shielding that requires interactions with neighboring glycans (21). In individuals that harbor virions with immunogenic bNAb epitopes, loss or shifts of glycan positions associated with escape from strain-specific antibodies might serve to modulate binding and neutralization by bNAbs (20, 22). Whereas N332 is central to potent bNAbs and broad serum responses (2, 5, 14, 16, 23–27), the epitopes of other glycan-dependent Nabs with lesser breadth have been found to include potential N-glycosylation site (PNGS) contacts at 276, 295, 334, 339, and 392 (19, 28–33) that would also be subject to immune selection.

In this study, we sequenced envelope (*env*) recovered from a single time point for seven individuals with rare outcomes to HIV-1 infection, including two normal-progressing elite neutralizers (ENs) and five slow-progressing viremic controllers (VCs). We tested each Env for sensitivity to known broadly neutralizing monoclonal antibodies (bN-mAbs) targeting the V2, V3-C3, and CD4bs and determined whether certain glycans were associated with greater median sensitivity to bN-mAbs (lower IC_50_s) when absent. We then examined whether those glycans were under positive selection in the individual viral populations. Although simple loss of a PNGS might confer escape from a glycan-dependent Nab, a signal of positive selection could indicate that glycan loss for immune escape conflicted with loss of shielding of an immunogenic bNAb epitope. Our results show that this scenario characterized the viral populations of the two ENs, but not the five controllers.

## RESULTS 

### Neutralizing antibody breadth

We estimated the level of cross-reactive neutralizing activity in sera from seven individuals (Table 1) for a standard panel of five viruses from four clades (Simek) used to define elite neutralizers (1). Two sera, including EN Clade B (previously described as EN1 [34]) and EN Clade AE (previously described as T500107 [35]), met the formal definition of breadth for ENs by neutralizing all five viruses at dilutions >1:300. The five other clade B subjects did not achieve the levels of breadth characteristic of an EN. VC1 sera neutralized the clade A and B viruses, but VC2 sera did not effectively neutralize any of the Simek panel viruses. The sera from VC3 (previously described as EN3 [36]) only neutralized the virus representative of clade A. VC4 sera neutralized the four viruses from clades A, B, and C, but not AE. The sera from VC5 neutralized the clade B and both clade C viruses.

**TABLE 1:** Serum neutralization titers of Simek panel viruses^*a*^

Participant	Virus source	No. of viruses	Simek panel neutralization titers (ID_50_ 1/dilution)					No. of viruses neutralized at >1:300 dilution
			94UG103 Clade A	92BR020 Clade B	93IN905 Clade C	M-C-026 Clade C	92TH021 Clade AE	
EN Clade B	Plasma	10	**785**	**1,241**	**986**	**507**	**408**	5
	PBMC	11						
VC1 (Clade B)	Plasma	10	**1,292**	**1,282**	227	34	37	2
	PBMC	10						
VC2 (Clade B)	Plasma	10	136	221	160	76	128	0
	PBMC	10						
VC3 (Clade B)	Plasma	0	**302**	249	81	27	65	1
	PBMC	20						
VC4 (Clade B)	Plasma	10	**377**	**365**	**900**	**754**	165	4
	PBMC	10						
VC5 (Clade B)	Plasma	10	120	**404**	**828**	**415**	20	3
	PBMC	10						
EN Clade AE	Plasma	31	**405**	**1,125**	**2,297**	**350**	**3,315**	5
	PBMC	0						

^*a*^ Titers >1:300 are in bold type. The two participants whose sera neutralized all five sequences from four clades are highlighted in gray.

### Viral phylogenies and genetic diversity estimates

Maximum likelihood (ML) trees were reconstructed for fully functional *env* recovered from all seven individuals (see Fig. S1A through G at https://doi.org/10.6084/m9.figshare.29534318.v1), five of which consisted of both ancestral proviral (37) and circulating plasma *env* sequences (see Fig. S1A, B, C, E, F at https://doi.org/10.6084/m9.figshare.29534318.v1). Only proviruses were recovered from VC3 (see Fig. S1D at https://doi.org/10.6084/m9.figshare.29534318.v1) and only plasma viruses were obtained from EN Clade AE (see Fig. S1G at https://doi.org/10.6084/m9.figshare.29534318.v1).

Levels of viral genetic diversity exhibited considerable differences among individuals (see Table S1 at https://doi.org/10.6084/m9.figshare.29534318.v1). Consistent with the reduced viral replication expected in HIV-1-infected people that maintain low viral loads (38), controllers in this study had lower levels of polymorphism in both their proviral and plasma virus populations. However, the plasma and proviral sequences in VC5 had the second highest amount of polymorphism observed, falling only behind EN Clade B. For individuals with both proviral and plasma sequences, the levels of nucleotide diversity (θπ) were strongly affected by the divergence between the two groups. Absolute divergence between plasma and proviral sequences (D_XY_) varied from 0.0153 in VC1 to 0.0856 in VC4 (see Table S1 at https://doi.org/10.6084/m9.figshare.29534318.v1). Tajima’s D was commonly negative, indicating an excess of rare and singleton mutations. Within individuals, the levels of polymorphism in the plasma and proviral sequences were strongly correlated (Pearson’s r = 0.842) but were not statistically significant due to VC2 being an outlier (*P* = 0.073).

Phylogenetic trees for each participant were annotated for the absence of commonly observed Env PNGS (see Fig. S1A through G at https://doi.org/10.6084/m9.figshare.29534318.v1). Viruses from four individuals, including EN Clade B (see Fig. S1A at https://doi.org/10.6084/m9.figshare.29534318.v1), VC1 (see Fig. S1B at https://doi.org/10.6084/m9.figshare.29534318.v1), VC4 (see Fig. S1E at https://doi.org/10.6084/m9.figshare.29534318.v1), and VC5 (see Fig. S1F at https://doi.org/10.6084/m9.figshare.29534318.v1) were fixed for N332, and viruses from VC3 (see Fig. S1D at https://doi.org/10.6084/m9.figshare.29534318.v1) were exclusively N334. EN clade AE viruses (see Fig. S1G at https://doi.org/10.6084/m9.figshare.29534318.v1) exhibited polymorphism of N334 as well as N392 and N465. Viruses isolated from VC2 (see Fig. S1C at https://doi.org/10.6084/m9.figshare.29534318.v1), whose sera did not neutralize any of the Simek panel viruses (Table 1), exhibited a glycan shift from N334 in 10 proviruses to N332 in 8/10 plasma viruses.

### Tests for positive selection

Fitting PAML models M7 and M8 (39) to the data, positive selection was observed in EN Clade B, EN Clade AE, VC1, VC2, and VC5 but not in VC3 or VC4 (see Table S2 at https://doi.org/10.6084/m9.figshare.29534318.v1). In EN Clade B and VC5, positive selection was detected in the proviral sequences, the circulating plasma sequences, and in the combined total sample. The strength of selection in EN Clade B (57 positively selected codons) and VC5 (26 positively selected codons) far exceeded that observed in other participants. Viral sequences from EN Clade B, EN Clade AE, and VC5 also had high levels of nucleotide diversity (and hence denser ML trees). However, the long internal branches in VC4 separating the proviral and plasma sequences (see Fig. S1E at https://doi.org/10.6084/m9.figshare.29534318.v1) produced the highest overall nucleotide diversity among individuals (θ_Π_= 0.0464) but no signal of positive selection.

Positive selection at N-X-S/T residues affecting glycan formation was detected in the V5 domain (N462 and N465) of the EN Clade AE plasma viruses, and in the C2 domain (N295), the C3 domain (N337, N339), the CD4-binding loop (N362), the V4 domain (N392) and the C4 domain (N444) of the EN Clade B plasma and proviruses. For the controllers, analysis of the combined plasma/proviral data sets showed positive selection at a residue associated with a polymorphic glycan in VC1 (N463) and in VC2 (N444). (Table 2).

**TABLE 2:** Positively selected sites by domain^*a*^

Env regions	SS	C1	V1	V2	C2/loop D	V3	C3	CD4 binding loop	V4	C4/β20β21/ β23	V5/β24	β24/C5	gp41
Antibody contact regions		CD4bs VRC01		PG9/PG16	CD4bs VRC01 2G12	PGT121/PGT128	PGT121/PGT128 2G12	CD4bs VRC01	2G12	CD4bs VRC01	CD4bs VRC01	CD4bs VRC01	
EN Clade B Provirus	(0)	87*	132* 134* (7)	165* 187**	268*	328*	335* 337** 339** 340** 347** 360*	362** 364**	392** (2)	(0)	(0)	(0)	(6)
EN Clade B Plasma virus	6*	92+*	133** 135** (7)	178* 179** 187**	200* 240** 273** 295** 297**	(0)	333** 335** 341** 343** 347** 350**	363* 364**	393* 413** (4)	444** 446*	(1)	471*	(4)
VC1	(0)	(0)	(0)	(0)	(0)	(0)	(0)	363*	(0)	(0)	464*	(0)	(1)
VC2	(0)	49*	(0)	(0)	(0)	(0)	(0)	(0)	(0)	446*	(0)	(0)	(2)
VC3	(0)	(0)	(0)	(0)	(0)	(0)	(0)	(0)	(0)	(0)	(0)	(0)	(0)
VC4	(0)	(0)	(0)	(0)	(0)	(0)	(0)	(0)	(0)	(0)	(0)	(0)	(0)
VC5	(0)	(0)	(8)	189**	292** 295**	322*	340* 354**	(0)	(1)	(0)	464*	(0)	(10)
EN Clade AE	(0)	85*	(0)	(0)	(0)	(0)	(0)	(0)	396*	442*	462** 465*	(0)	(1)

^*a*^ Positively selected sites for EN Clade B plasma and provirus sample as determined for Table S2 (https://doi.org/10.6084/m9.figshare.29534318.v1). Positively selected sites for remaining participants shown for analysis of combined plasma/proviral data sets. Positively selected residues are indicated as ** for *P* > 0.99 or * for *P* > 0.95. Positions are denoted by HXB2 numbering for ordered regions of gp120 or by the number of selected sites (#) in disordered regions and in gp41. SS refers to the signal sequence domain. Sites affecting the formation of a PNGS (N-X-S/T) are in bold type. Residue 471 (EN Clade B) is a CD4bs contact. Residues 462 and 465 (EN Clade AE) are VRC01 contacts.

### Trends in bN-mAb neutralization sensitivity associated with absent glycan

For the combined virus sample, medians of bN-mAb titers for the presence and absence of select PNGS were compared by Mann-Whitney U tests (Table 3). The absence of N295, N334, N339, N413, N392, and N465 was found to be associated with lower median IC_50_ values (greater median viral sensitivity) to various bN-mAbs. However, only the absence of N295, N392, and N465 associated with lower IC_50_s of the CD4-binding site (CD4bs) directed bNAb, VRC01 (40). The absence of the same three PNGS also correlated with lower median IC_50_ values of both an N332 bNAb (PGT121 [25]) and the V2 bNAbs, PG9, and PG16 (41). The 332 PNGS are central to the epitopes of PGT121 and PGT128 (23, 25, 30) and, as expected, the presence of N332 was associated with their significantly lower median IC_50_ values. When missing, N339 correlated with lower median IC_50_ values for PG9, PG16, and PGT121. The lack of N413 correlated only with greater sensitivity to PG9, but the presence of N413 and N295 correlated with greater sensitivity to PGT128. The presence of N289 correlated with greater sensitivity to VRC01, PG9, and PG16. The V5 PNGS, N462 and N463, were not tested due to the strain-specific nature of the alignments. N337 and N444 were not tested due to low variance in occurrence.

**TABLE 3:** Median bN-mAb neutralization titers (IC_50_ µg/mL) for the presence and absence of select PNGS^*a*^

	Feature	N289	N295	N332	N334	N339	N413	N392	N465
**VRC01**	Present	(**114) 1.33**	(100) 2.59	(**88) 1.36**	(51) 1.79	(82) 1.90	(61) 1.79	(98) 3.68	(57) 3.67
	Absent	(38) 7.79	(**52) 0.62**	(64) 2.16	(101) 1.76	(70) 1.70	(91) 1.76	(**54) 0.65**	(**95) 0.87**
	*P* Value	**0.0055**	**<0.0001**	**0.0083**	0.6581	0.0707	0.2192	**<0.0001**	**<0.0001**
**PG9**	Present	(**114) 0.77**	(100) 25.0	(**88) 0.58**	(51) 25.0	(82) 25.0	(61) 25.0	(98) 22.0	(57) 25.0
	Absent	(38) 25.0	(**52) 0.47**	(64) 25.0	(**101) 0.74**	(**70) 0.40**	(**91) 0.43**	(**54) 0.34**	(**95) 1.39**
	*P* Value	**<0.0001**	**0.0001**	**<0.0001**	**0.0015**	**<0.0001**	**<0.0001**	**<0.0001**	**0.0012**
**PG16**	Present	(**104) 1.95**	(90) 25.0	(**88) 0.75**	(43) 25.0	(72) 25.0	(61) 1.79	(89) 25.0	(49) 25.0
	Absent	(38) 25.0	(**52) 0.70**	(54) 25.0	(**99) 1.39**	(**70) 1.033**	(81) 10.65	(**53) 1.10**	(**93) 1.39**
	*P* Value	**0.0193**	**<0.0001**	**<0.0001**	**<0.0001**	**0.0083**	0.7342	**0.0004**	**0.0007**
**PGT128**	Present	(114) 0.76	(**100) 4.12**	(**88) 0.68**	(51) 20.0	(82) 20.0	(**61) 0.04**	(98) 16.2	(57) 0.47
	Absent	(38) 9.64	(52) 25	(64) 25.0	(101) 2.54	(70) 2.12	(91) 20	(54) 0.51	(95) 13.8
	*P* Value	0.0654	**0.0319**	**0.0264**	0.8495	0.2165	**0.0020**	0.8082	0.0053
**PGT121**	Present	(103) 25.0	(89) 25.0	(**88) 1.11**	(42) 25.0	(71) 25.0	(61) 25.0	(88) 25.0	(48) 25.0
	Absent	(38) 3.84	(**52) 10.66**	(53) 25.0	(**99) 1.52**	(**70) 4.36**	(80) 25.0	(**53) 1.37**	(**93) 6.52**
	*P* Value	0.1591	**0.0372**	**<0.0001**	**<0.0001**	**0.0004**	0.52	**0.0010**	**0.0102**

^*a*^ IC_50_ values represent the medians of data from all seven individuals. Numbering of glycan positions is HXB2. Total numbers of virus with present/absent glycans given as (N). As determined by Mann-Whitney U test, significantly lower IC_50_ values corresponding to the presence or absence of a PNGS are noted in bold type. The combined virus samples totaled 152, but we lacked PG16 data for 10/31 EN Clade AE viruses, making N = 142 for the PG16 data set. Similarly, the data set for PGT121 was N = 141 as we lacked data for 11/31 Clade AE viruses.

### Viral evolution in EN Clade B, VC1 and VC2

**Fig 1:**
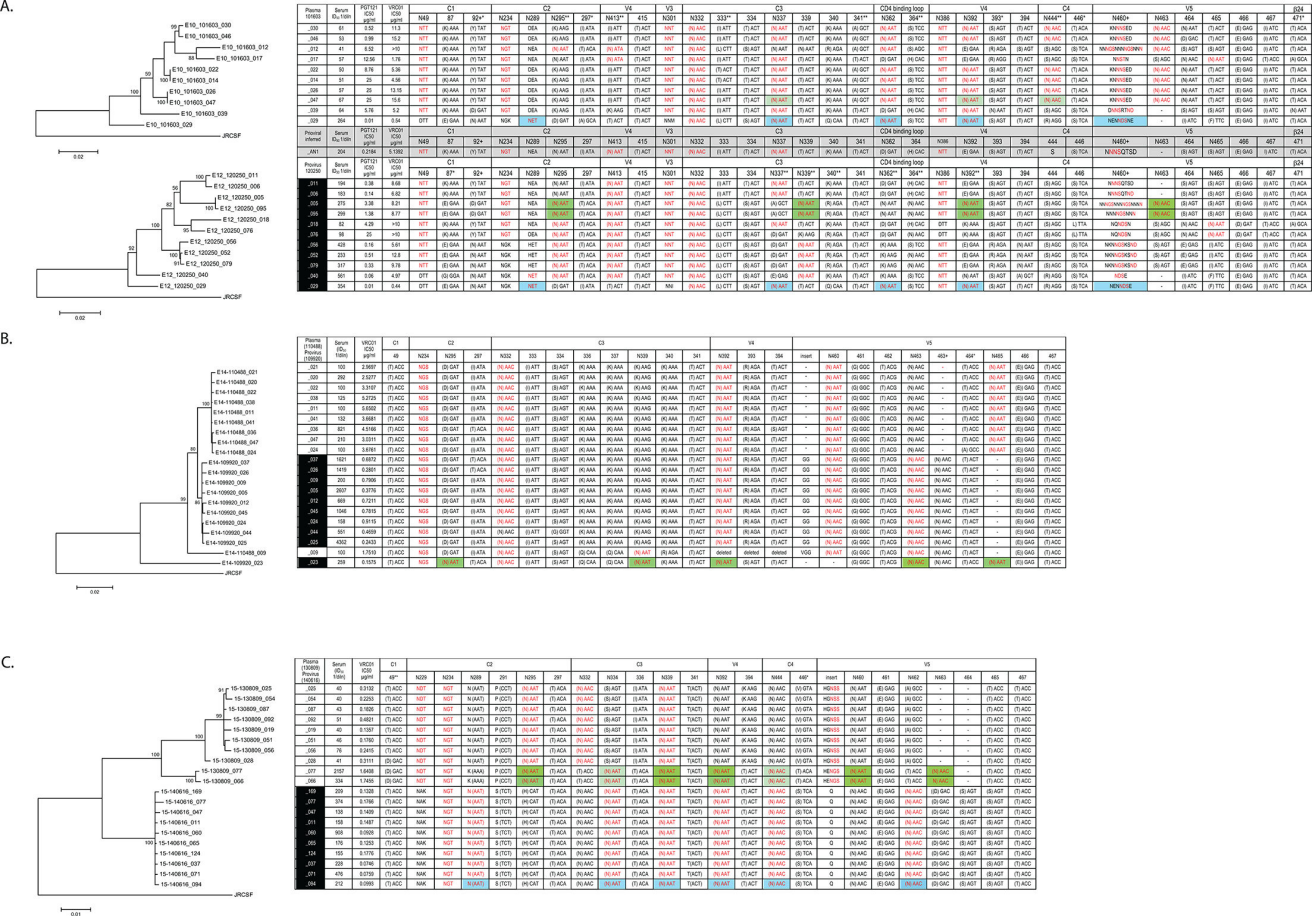
Viral evolution in (**A**) EN Clade B, (**B**) VC1, and (**C**) VC2. (A) Individual ML trees are shown for the EN Clade B plasma and provirus samples. An inferred proviral sequence (previously described as AN1 [34]) is highlighted in gray. An N289-N337-N362-N392-N460+ combination is highlighted in blue. An N295-N339-N392-N463 combination is highlighted in dark green. An N337-N392-N444 combination is highlighted in light green. (B) The VC1 ML tree was inferred from the combined plasma/provirus sample. An N295-N339-N392-N463-N465 combination is highlighted in dark green. (C) The VC2 ML tree also represents the combined plasma/proviral sample. An N289-N334-N339-N392-N444-N462 combination is highlighted in blue. An N295-N339-N392-N460-N463 combination (also present in EN Clade B) is highlighted in dark green, and N334-N444 is highlighted in light green. For viruses from all three participants, positively selected residues are indicated as ** for *P* > 0.99 or * for *P* > 0.95. Provirus IDs are shaded black and plasma virus IDs are unshaded. The first position in a complete PNGS sequon (N-X-S/T) is in bold, red font.

EN Clade B viruses showed evidence of a polyclonal immune response. Multiple positively selected sites were detected in the C3 domain (Table 2), which is consistent with HIV-1 population samples from other sera exhibiting cross-reactivity (42). Positive selection detected at position 333 of the N332 glycosylation sequon in the plasma virus sample was suggestive of an N332 specificity but with retention of ^332^N-X-S/T^334^ in all viruses (Fig. 1A). Positive selection at CD4bs-related contacts, including residues 471, 362, and 364 (Table 2), pointed to a co-occurring CD4bs specificity. Amino acids at 362 and 364 determined the sequon for N362, a PNGS that lines the CD4bs cavity (43), and position 471 is a CD4bs contact in β24 (40, 44).

The viruses that anchored the EN Clade B plasma and proviral ML trees showed the greatest sensitivity to both PGT121 and VRC01, with both displaying an N289-N337-N362-N392-N460 glycan combination (Fig. 1A). Whereas polymorphism of N392 (in combination with N332) might suggest the presence of a PGT135-like antibody (30) where loss of either N332 or N392 is required for viral escape (20, 30, 31), positive selection at residues affecting formation of N295, N339, and N392 (Table 2) was suggestive of selection by a 2G12-like glycan-dependent antibody that requires loss of N295 and N392 for escape (28, 29). A complete 2G12-like epitope appeared in two mid-tree proviruses in combination with a V5 PNGS (N463) (Fig. 1A). Thereafter, the loss of N295, the introduction of N444, and a shift of N339 to N337 created a new glycan combination (N337-N392-N444) with positive selection detected at N444.

In addition, N49 (C1) and N234 (C2) were introduced to the EN Clade B proviral population and carried through to 9/10 plasma viruses. V1 loop lengths for the provirus ranged from 25 to 39 amino acids compared to 26–27 residues for the plasma virus. However, the V2 loop lengths of the serum-resistant plasma viruses increased by 5–14 residues compared to the proviruses.

For VC1, whose sera neutralized only 2/5 Simek panel viruses (Table 1), a PNGS combination comprising a 2G12-like epitope plus two V5 glycans (N463, N465) was observed for a single provirus at the base of its ML tree (Fig. 1B). Thereafter, absent signals of positive selection, N295 disappeared from 19/20 viruses, N339 was lost from 18/20 viruses, and N392 was deleted from a single plasma virus. N465 was also lost from 9/10 proviruses but reappeared in 9/10 plasma viruses accompanied by the loss of N463. In addition to the observed glycan polymorphism, a 13-amino acid insertion increased the V1 loop length of all VC1 plasma viruses to 46 residues, which corresponded to a reduction in autologous serum neutralization titers and decreased viral sensitivity to VRC01.

The VC2 viral population, which did not induce cross-reactive responses (Table 1), exhibited a basal glycan combination of N289-N334-N339-N392-N444-N462 (Fig. 1C). The loss of N289 and N462 was accompanied by the introduction of N295 which formed a 2G12-like plus V5 glycan combination (N295-N339-N392-N460-N463) in two plasma viruses. Thereafter, the loss of N392, N444, N460, and N463, plus a shift of N334 to N332, coincided with serum neutralization resistance. Only the loss of N444 was accompanied by a signal of positive selection at residue 446 (Fig. 1C). The VC2 proviruses had V1 loop lengths of 26 residues and the plasma virus ranged from 23 to 29 amino acids.

### Viral evolution in EN Clade AE, VC3, and VC4

**Fig 2:**
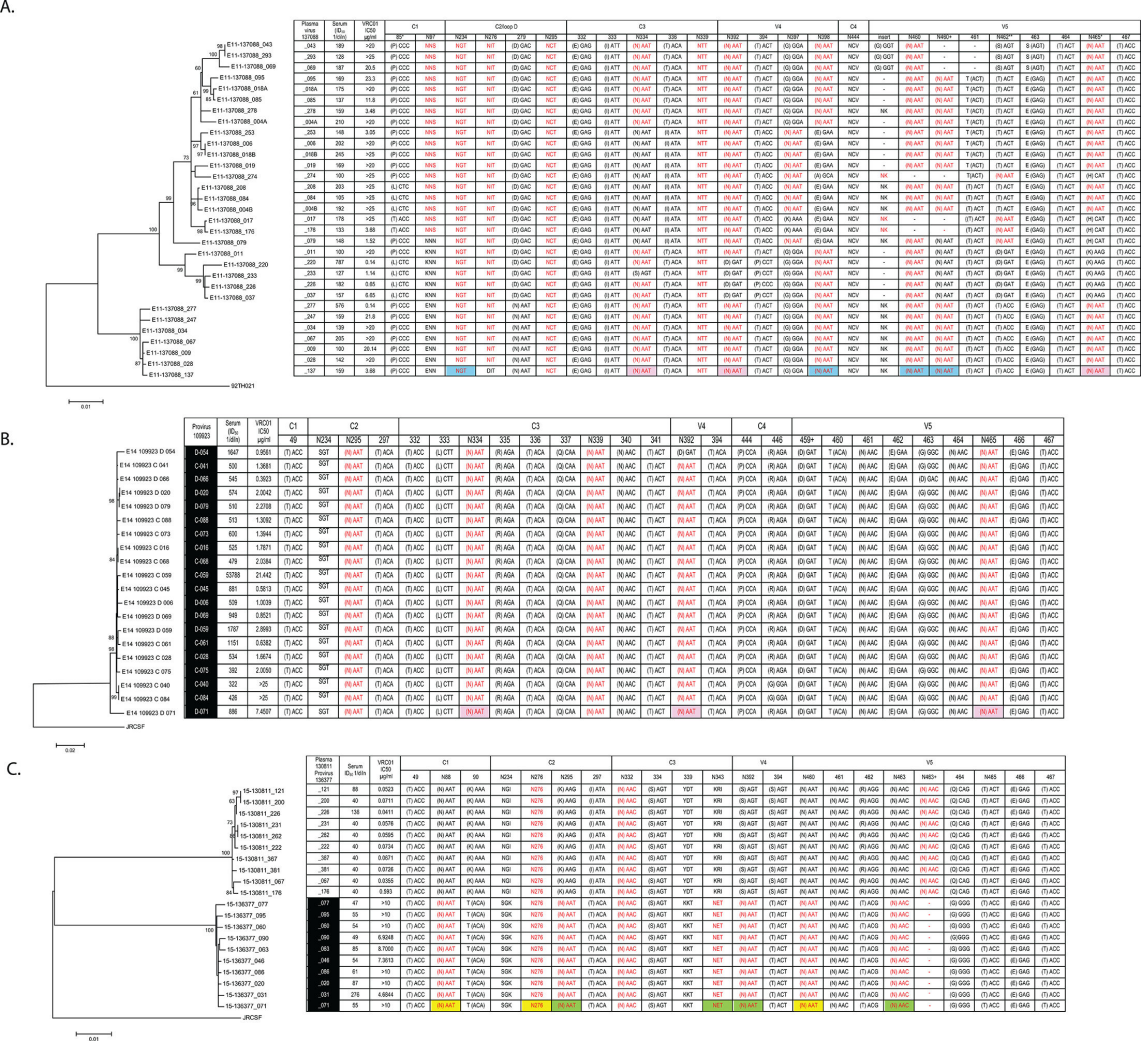
Viral evolution in (A) EN Clade AE, (B) VC3, and (C) VC4. (A) EN Clade AE plasma viruses. The PNGS combination N334-N392-N465 is shaded pink, N234-N398-N460/N460+ is shaded blue. Positively selected residues are indicated as ** for *P* > 0.99 or * for *P* > 0.95. (B) VC3 proviruses. The PNGS combination N334-N392-N465 is shaded pink. (C) VC4 plasma/proviruses. The PNGS combination N88-N276-N460 is shaded yellow, and N295-N343-N392-N463 is shaded green. Complete PNGS sequons (N-X-S/T) are shown in either red font or designated by red font of the first residue (N).

EN Clade AE viruses exhibited bNAb epitope mutations suggestive of selection by a VRC01-like antibody. Positive selection detected at glycan positions 462 and 465 (Fig. 2A, Table 2) corresponded to a VRC01 contact region in the V5-β24 domain (45). N465, which had correlated with lower median sensitivity to VRC01 when present (Table 3), was absent from 9/31 viruses. The N-X-S/T sequon for N465 encompassed VRC01 contact residues at 465 and 467 (40, 44, 46). Further VRC01 epitope mutations included the introduction of a PNGS at HXB2 position 97 (with N97 replacing K97, a VRC01 contact in the C1 domain [40]), and the mutation of a VRC01 contact in loop D. The latter mutation changed the ancestral N279 to D279 and corresponded to the split of the EN Clade AE plasma viruses into two clades (Fig. 2A; see Fig. S1G at https://doi.org/10.6084/m9.figshare.29534318.v1). We found the median VRC01 IC_50_ for all viruses with N279 was 0.501 µg/mL (*N* = 42), which was significantly more sensitive than 3.042 µg/mL (*N* = 110) for viruses with D279 (*P* = 0.015). Only the basal virus of its ML tree lacked N276. All lower clade viruses exhibited V1 loop lengths of 28 amino acids, and the upper clade viruses varied between 28 and 35 residues. The V2 loop length of the lower clade viruses was 36 residues versus 38–42 amino acids for the upper clade.

The EN Clade AE Env mutations implicating selection by a VRC01-like specificity occurred in concert with polymorphism of PNGS suggestive of co-occurring selection by glycan-dependent antibodies. Putative glycan epitopes in the basal virus of its phylogeny included N234-N398-N460/N460+ and N334-N392-N465 (Fig. 2A) with the latter combination reflecting a C3/V5-style epitope described for BG505 (47).

VC3 sera neutralized only 1/5 Simek panel viruses (Table 1) despite high autologous serum neutralization titers ranging from 322 to 53,788 (ID_50_ 1/diln) for individual viruses (Fig. 2B). Like EN Clade AE, we observed that an N334-N392-N465 PNGS combination was present in 19/20 proviruses (Fig. 2B). However, only a single virus exhibited loss of N392 (without a signal of positive selection). The V1 loop length of the basal VC3 provirus was 37 amino acids versus 35 residues in the remaining viruses.

All viruses from VC4, whose sera had neutralized 4/5 Simek panel viruses, lacked N465 (Fig. 2C). Putative glycan epitopes present in the provirus anchoring the VC4 ML tree included a C1-C2-V5 glycan combination (N88-N276-N460) and a C2-C3-V4-V5 glycan combination (N295-N343-N392-N463). N88, N295, N343, N392, N460, and N463 were lost from the plasma viruses with N463 shifting to N463+. Unusually, V1 lengths of the VC4 proviruses exceeded that of the plasma viruses (38 versus 28 residues). The proviral to plasma virus glycan losses and decrease in V1 loop lengths corresponded to a 79-fold increase in the average VRC01 neutralization sensitivity of the plasma viruses (8.77–0.11 µg/mL). However, all autologous serum neutralization titers were less than 300 (ID_50_ 1/diln).

### Viral evolution in donor 45, VC5, and CH505

Positively selected glycan variation was also detected for the clade B *env* sequences recovered from the donor 45 individual from which VRC01 was first isolated (48). Positive selection was detected at residues affecting formation of N49 (C1), N335 (C3), and N444 (C4) (Table 4). Positive selection was also detected at six other C1 residues including the VRC01 contact at HXB2 97, and loop D residues 277 (the central amino acid of the N276 sequon), 279, and 281. The effects on VRC01 neutralization sensitivity corresponding to the positively selected loop D residues 279 and 281 were previously described (46).

**TABLE 4:** Positively selected sites by domain for donor 45 and CH505^*a*^

Participant	SS	C1	V1	V2	C2/loop D	V3	C3	V4	C4	V5	C5	gp41
Donor 45	0	33** 34** 49** 62** 87** 95* 97*	(4)	161** 162** 169* 190** (2)	238** 274** 277** 279** 281** 290*	308** 317* 318** 321**	336** 337** 343* 344** 352**	412**	436* 440** 442** 444*	0	496* 500**	(18)
CH505	0	0	(1) 152*	0	281**	325* 330*	347** 355**	397* 398** 416** 417**	0	0	0	(4)

^*a*^ Positions are denoted by HXB2 numbering for ordered regions of gp120 or by the number of selected sites (#) occurring in disordered regions and in gp41. Sites affecting formation of a PNGS (N-X-S/T) are in bold type. Residues 97 (C1), 279, and 281 (loop D) are VRC01 contacts. Positively selected PNGS are indicated as ** for *P* > 0.99 or * for *P* > 0.95.

**Fig 3:**
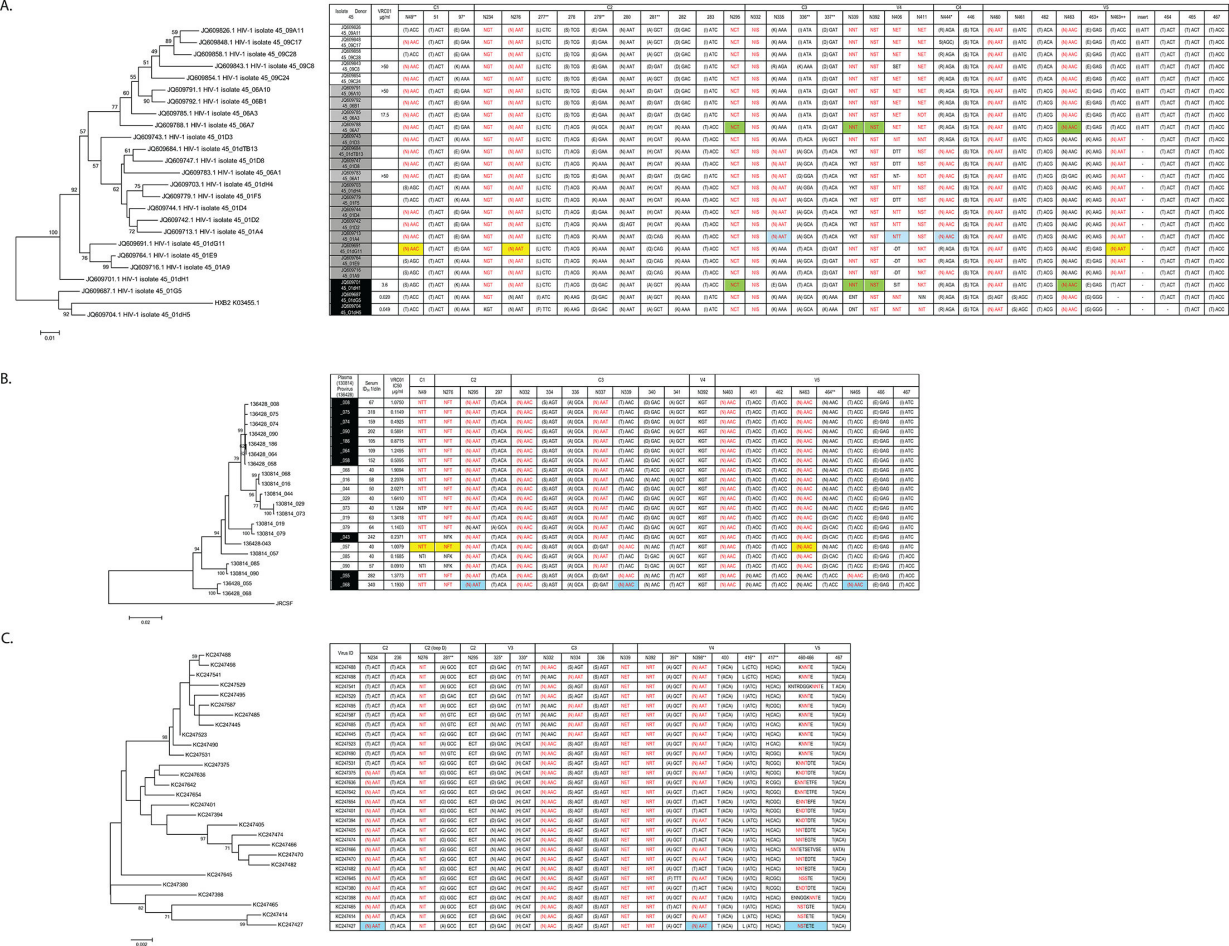
Viral evolution in (**A**) donor 45, (**B**) VC5, and (**C**) CH505. (A) VRC01 donor subject (donor 45). Viruses are in the tree order. Proviruses recovered from 2001 are shaded black. Plasma viruses recovered from 2001 (dark gray), 2006 (light gray), and 2009 (white). The PNGS combination N295-N339-N392-N463 is shaded green. N49-N276-N463 ++ is shaded yellow. N335-N406-N444 is shaded blue. (B) VC5 viruses are in the tree order. Proviruses are shaded black, and plasma viruses are unshaded. The PNGS combination N295-N339-N465 is shaded blue, and N49-N276-N463 is shaded yellow. (C) CH505 viruses are in the tree order. The glycan combination N234-N398-V5 is shaded blue. Complete PNGS sequons (N-X-S/T) are shown in either red font or designated by red font of the first residue (N). Positively selected residues are indicated as ** for *P* > 0.99 or * for *P* > 0.95.

A ML tree of select donor 45 viruses (Fig. 3A) was anchored by a VRC01-sensitive provirus (45_01dH5) lacking N234, N276, and N339. The V1 and V2 lengths of the basal virus were 24 and 39 amino acids, respectively. A 73-fold reduction in proviral (45_01dH1) sensitivity to VRC01 (0.049–3.6 µg/mL) corresponded to the appearance of N234, N276, and N339 plus an increase in the V1 and V2 loop lengths to 51 and 45 residues, respectively. The introduction of N339 formed an N295-N339-N392-N463 glycan combination (2G12-like plus V5 PNGS) that was also seen in EN Clade B (Fig. 1A). The introduction of N49 and N276 formed a C1-C2-V5 glycan combination (N49-N276-N463++) that was like a polymorphic N49-N276-N465 glycan combination observed in VC5 viruses (Fig. 3B). The introduction of N444 to donor 45 viruses formed a polymorphic N335-N406-N444 glycan combination with the occurrences of N335 and N339 being mutually exclusive. All donor 45 viruses exhibited ^465^TET^467^ in V5/β24.

For the longitudinally sampled viruses from the CH505 clade C viral population (9), positive selection was detected at residue 281 in loop D, residues 325 and 330 in the V3, and residues 398, 416, and 417 in the V4 (flanking the β20-β21 domain) (Table 4). The loop D mutations that drove development of a CD4bs bNAb via overlapping CD4bs Nabs were previously described (12). CH505 viruses exhibited polymorphism of N234, N332, N334, N398, and of V5 PNGS (Fig. 3C). Polymorphism of an N234-N398-N460 PNGS combination reflected a similar glycan combination present in EN Clade AE viruses (N234-N398-N460/N460+). V1 loop lengths varied between 16 and 32 residues.

### Immune responses elicited to VRC01, V2, and V3/C3 epitopes

**Fig 4:**
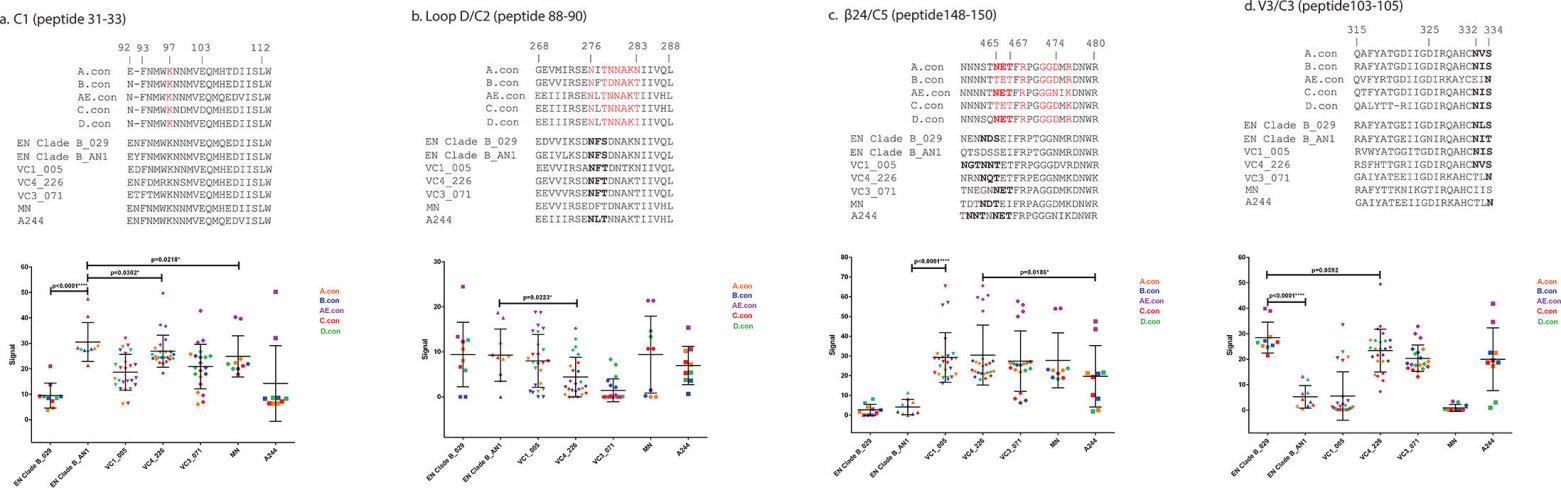
Positivity responses to consensus peptides overlapping the VRC01 epitope. (**a**) C1, (**b**) Loop D/C2, (**c**) β24/C5, and the N332 epitope (**d**) V3/C3. Consensus peptides are annotated for VRC01 contacts (red type). PNGS are in bold type.

Positive selection detected at PNGS in the EN viral populations was suggestive of underlying immunogenic residues. We compared the regional cross-reactive immune responses elicited to VRC01, V2, and V3/C3 epitopes by similarly designed recombinant gp120 antigens in rabbit immunizations using a peptide assay developed and conducted at Duke University (49). The means of positivity responses (Table 5) for associated peptide/antigen sequence (Fig. 4a through d; see Fig. S2a through e at https://doi.org/10.6084/m9.figshare.29534318.v1) represented the sum of positivity responses for three consensus peptides overlapping bNAb epitope positions for clades A, B, AE, C, and D.

**TABLE 5:** Means and standard deviations (in parentheses) for positivity responses to consensus peptides overlapping VRC01, V2, and V3/C3 bNAb epitope contacts for clades A, B, C, D, and AE

bNAb epitope	Peptide	Env domain	Antigen						
			EN Clade B-029	EN Clade B-AN1	VC1-005	VC4-226	VC3-071	MN	A244
VRC01	31–33	C1	9.49 (4.89)	30.54 (7.64)	18.66 (7.04)	26.94 (6.31)	20.89 (8.73)	24.91 (8.11)	14.24 (14.85)
VRC01	39–41	C1/V1	7.53 (2.62)	4.36 (2.87)	6.19 (2.60)	6.86 (4.33)	7.90 (5.34)	13.28 (5.32)	2.32 (5.13)
VRC01	88–90	Loop D	9.40 (7.16)	9.28 (5.80)	7.98 (5.86)	4.39 (4.41)	1.45 (2.54)	9.38 (8.54)	6.96 (4.24)
VRC01	117–119	CD4-binding loop	2.70 (5.66)	1.48 (2.16)	6.72 (10.22)	5.32 (7.69)	3.22 (5.43)	11.01 (10.46)	9.57 (11.73)
VRC01	134–136	β20/β21	5.71 (6.41)	8.17 (7.34)	1.61 (3.42)	4.18 (5.75)	1.42 (2.07)	4.08 (5.41)	4.08 (6.73)
VRC01	144–146	β23/V5/ β24	8.25 (7.01)	8.22 (9.03)	11.55 (4.93)	9.02 (6.63)	9.23 (5.88)	13.28 (9.91)	15.64 (7.21)
VRC01	148–150	β24/C5	2.66 (2.76)	4.09 (3.83)	29.2 (12.59)	30.43 (15.23)	27.36 (15.32)	27.76 (13.96)	19.69 (15.57)
V2	54–56	V2	12.09 (7.46)	2.07 (4.54)	4.87 (5.22)	0.79 (0.89)	1.33 (1.60)	2.64 (2.68)	4.89 (6.61)
V3/C3	103–105	V3/C3	28.49 (6.08)	5.22 (4.46)	5.51 (9.49)	23.38 (8.43)	20.35 (5.25)	0.88 (1.39)	19.96 (12.33)
No. of rabbits immunized			2	2	5	5	4	2	2
No. of data points			10	10	25	25	20	10	10

Viruses that were neutralization sensitive to both autologous serum and bN-mAbs were selected as antigens from EN Clade B, VC1, VC3, and VC4. In addition, for EN Clade B, we considered phylogenetic positioning for comparing the immunogenicity of a basally placed virus (120250_029) to an inferred sequence (AN1) (see Fig. S1A at https://doi.org/10.6084/m9.figshare.29534318.v1) located at the branch proximate to serum neutralization-resistant viruses (34). The AN1 antigen was less sensitive to autologous sera, VRC01, and PGT121 than the 029 antigen (Fig. 1A). For comparison, we also included data from similarly designed MN (clade B) and A244 (clade AE) constructs. Regarding PNGS associated with increased sensitivity to VRC01 when absent (Table 3), N465 was present in the VC3 and A244 antigens. N392 was absent from the EN Clade B_AN1 antigen but present in 029. N392 was also absent from VC4 and MN but present in VC1, VC3, and A244. N295 was present in VC3, MN, and A244 but absent from the EN Clade B and VC4 antigens.

The strongest positivity responses overlapping VRC01 epitope contacts were seen for the C1 and β24/C5 peptides. The C1 peptides (Fig. 4a) encompassed the VRC01 contact at residue 97. All immunogens were K97, but the EN Clade B_AN1 antigen with Y92+ and a PNGS at HXB2 49 (Fig. 1A) elicited a significantly greater response than the EN Clade B_029 antigen, which was N92+ and lacked N49. The median response elicited by AN1 was also significantly greater than that of the three controllers, MN and A244 (VC1_005: *P* = 0.0004***; VC4_226: *P* = 0.0302**; VC3_071: *P* = 0.0015**; MN: *P* = 0.00218*; A244: *P* = 0.0136*). The VC4 construct, which lacked N88 (Fig. 2C) and exhibited D94 (Fig. 4a), stimulated a significantly higher median C1 response than the VC1 (*P* = 0.002***), VC3 (*P* = 0.0169*), and MN (*P* = 0.0180*) constructs which displayed N88 and N94 (residue).

Both EN Clade B antigens with ^465^SEI^467^ elicited negligible responses to the β24/C5 peptides (Fig. 4c) in comparison to the three immunogens derived from controller subjects with ^465^TET^467^ or ^465^NET^467^ (*P* < 0.0001). All three controller antigens possessed T467, which matched the residue present in the five consensus peptides. VC3 and A244, both of which were N465, showed the lowest ranges of observed responses, but VC3 exhibited a greater median response than A244 (*P* = 0.0392*). The median of the response elicited by the MN antigen with ^465^TEI^467^ was not significantly different from the controller antigens, suggesting the abrogated response was associated with S465. No significant differences were observed between the median responses of the controller antigens despite their differing mutations at position 471 (VC1 vs VC4, *P* = 0.7005; VC1 vs VC3, *P* = 0.4480; and VC4 vs VC3, *P* = 0.8299).

In contrast to the highly significant differences between responses elicited by select antigens to the C1 and β24/C5 peptides, responses to other portions of the VRC01 epitope including the C1/V1, CD4-binding loop, β20β21, and β23/V5/β24 (see Fig. S2a through d at https://doi.org/10.6084/m9.figshare.29534318.v1 ), and to the PG9/PG16 epitopes (V2) (see Fig. S2e at https://doi.org/10.6084/m9.figshare.29534318.v1) were weaker and more variable. Responses to peptides representing the loop D portion of the VRC01 epitope (Fig. 4b) were also relatively weak. However, both EN Clade B antigens and the VC1 antigen displaying N283 elicited greater median loop D responses than the VC3 and VC4 antigens with T283 but not greater than MN and A244 which were also T283 (MN vs 029: *P* = 0.8405; vs AN1: *P* = 0.8532; vs VC1: *P* = 0.7255), (A244 vs 029: *P* = 0.3424, vs AN1: *P* = 0.3930, vs VC1: *P* = 0.7001). Both EN Clade B antigens displayed an N276 PNGS as ^276^NFS^278^, but MN lacked N276 (^276^DFT^278^) and A244 had ^276^NLT^278^. An N279 (residue), which had corresponded to greater median VRC01 sensitivity than D279, was present in A244. The data for loop D illustrated that the median cross-reactive responses for antigens with N283 (029, AN1, VC1) or N279 and L277 (A244) were like an antigen lacking N276 (MN), and that the lowest median responses were elicited for the VC3_071 antigen displaying F277, N282, and T283.

For the N332 bNAb epitope (Fig. 4d), the EN Clade B_029 antigen with D325 and ^332^NLS^334^ exhibited a significantly greater response to the V3C3 peptides than AN1 with N325 and ^332^NIT^334^ (*P* < 0.0001) but not significantly different from the VC4_226 antigen with ^332^NVS^334^ and D325 (*P* = 0.0592). The V3 sequence motif ^324^GDIR^327^ is a component of four classes of glycan-bNAbs (50). The low median positivity response for VC1, which displayed D325 with ^332^NIS^334^, was not significantly different from the AN1 antigen with ^332^NIT^334^ (*P* = 0.0802). Two other antigens with D325 included VC3 and A244. Both the VC3 and A244 antigens, which lacked a N332 PNGS (T332) but had L333, elicited similar median response levels (*P* = 0.7457) that were not significantly different from the VC4 antigen displaying N332 and V333 (*P* = 0.0846 and *P* = 0.2547, respectively). However, the A244 responses were highly variable (Table 5). The responses elicited by the MN antigen, which also lacked an N332 PNGS (^332^IIS^334^) and displayed T325, were negligible. These data point to L333, as seen for the N332 sequon displayed by EN Clade B_029, as being an immunogenic, cross-reactive residue.

Overall, the basal virus of the EN Clade B phylogenetic tree (029) with L333 was more cross-reactive at the V3/C3 bNAb epitope than a later construct, but the AN1 antigen with Y92+ and an additional C1 PNGS (N49) was more cross-reactive in the C1 domain. Of the controllers, the VC4 antigen lacking N88, N295, and N392 elicited strong positivity responses to three bNAb epitope regions including the C1, β24/C5, and V3/C3.

### VRC01 sensitivity in relation to polymorphism of N465

The lower range of immune responses observed for two antigens exhibiting ^465^NET^467^ in the β24/C5 domain (VC3_071 and A244) (Fig. 4c) suggested a shielding role for N465. However, positive selection of N465 in the EN clade AE viral population (Fig. 2A) pointed to N465 as being a component of a glycan-dependent epitope in addition to shielding against a VRC01-like response. For EN Clade B, the sequence combination ^465^SEI^467^ was associated with an abrogated β24/C5 immune response, and the absence of N465 from 18/21 viruses in its phylogeny was associated with T467I mutations and mutation of the residue at 465. Whereas the EN Clade B plasma viruses were largely serum neutralization resistant, the single plasma virus (017) that retained ^465^NET^467^ exhibited sensitivity to VRC01 (<2 µg/mL) (Fig. 1A; see Fig. S1A at https://doi.org/10.6084/m9.figshare.29534318.v1).

For VC1, the absence of a PNGS at 465 in 9/10 proviruses occurred via the loss of the N465 residue, which then reappeared in the plasma viruses (Fig. 1B). Proviruses lacking an N465 PNGS were, on average, 5.5-fold more neutralization sensitive to VRC01 than the nine plasma viruses exhibiting N465 (0.70 vs 3.85 µg/mL). The single VC1 plasma virus lacking both N392 and N465 (110488_009) was twice as sensitive to VRC01 than the average of nine plasma viruses exhibiting the N392/N465 combination (1.75 vs 3.85 µg/mL). For VC5, N465 was present only in the two basal proviruses of its phylogeny (Fig. 3B). Thereafter, the absence of N465 was due to two proviruses having evolved an N465T mutation that also introduced N463. The presence of N463 and the evolution of a T467I mutation then carried through the remaining 16 VC5 viruses.

A survey of global viral glycan combinations representing either a 2G12-like epitope (N295-N339-N392) or a putative N334-based glycan epitope (N334-N392-N465) (Table 6) revealed 40% of clade AE viruses exhibited the N334-N392-N465 PNGS combination compared to <10% in clades A, B, C, and D. By contrast, the 2G12-like glycan combination was present in <10% of clades AE and C viruses versus >45% in clades B and D. Fewer than 20% of clade C viruses exhibited N295, and fewer than 7% exhibited N465. For both clades B and C, the frequency of I467 mutations exceeded the frequency of N465. In comparison, both N234 (C2 domain) and N392 (V4 domain) were conserved across clades at approximately 70% or higher. In the C1 domain, the highest occurrence of N49 (18.22%) was seen for clade B viruses, whereas N97 was not detected in any of the sequences examined.

**TABLE 6:** Percent occurrence of select PNGS and putative glycan epitopes by HIV-1 clade^*a*^

Clade	N49	N97	N234	N295	N332	N334	N339	N392	N295- N339- N392	T467	I467	N465	N334- N392- N465
A	0.87	0	78.35	47.19	69.17	29.87	65.37	80.52	28.14	77.5	17.6	49.35	8.23
B	18.22	0	71.12	79.86	84.95	10.25	70.61	83.31	48.54	54.2	36.4	18.79	1.22
C	1.92	0	85.95	18.45	78.35	12.06	68.32	69.57	7.98	52.2	43.5	6.47	6.39
D	11.3	0	82.61	73.91	79.13	8.7	74.78	78.26	46.09	90.4	8.8	62.6	3.45
AE	9.11	0	85.19	87.24	0.91	92.94	47.15	78.82	6.3	74.7	23.9	51.25	40.55

^*a*^ Percentage values were determined from sequences in the 2013 Los Alamos National Laboratory (LANL) database.

### Glycan placement in relation to the VRC01 epitope

**Fig 5:**
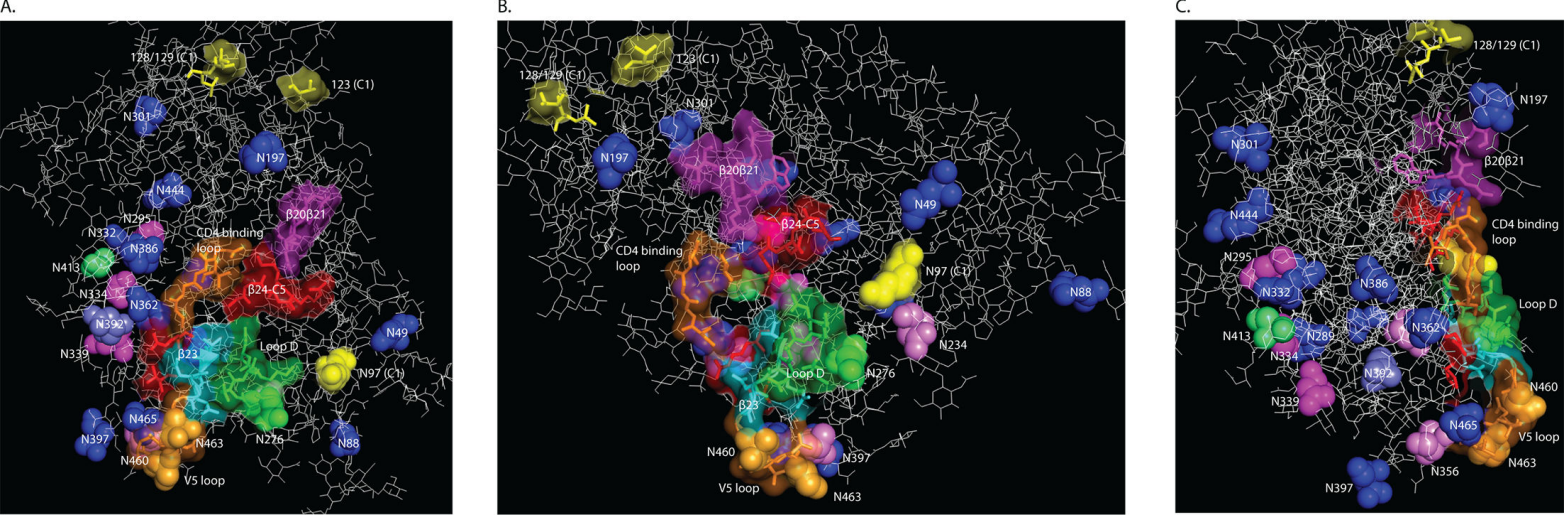
Spatial relationship of PNGS to the VRC01 epitope. (A) C1/C3 glycan view, (B) C1 glycan view, and (C) C3 glycan view. Numbering of glycan positions is HXB2. PDB:4TVP.

To understand how selection by glycan antibodies might affect neutralization sensitivity to VRC01, we examined the positioning of PNGS in relation to the VRC01 epitope (Fig. 5A–C), as mapped onto PDB:4TVP (BG505 trimer) (51). For clarity, we did not map every V5 PNGS observed in viruses of this study (i.e., N461 and N462), and N397 was a stand-in to show the relative placement of N398 due to the shortened V4 in PDB 4TVP. N397/N398 were polymorphic in EN Clade AE viruses (Fig. 2A), and N398 was polymorphic in the CH505 viral population (Fig. 3C).

PNGS in the C1 domain (N49 and N88), C2/loop D domain (N197, N234, and N276), CD4-binding loop (N362), V4 domain (N386, N392, and N397), and V5 domain (N460, N463, and N465) ring the VRC01 epitope (Fig. 5A). From a C1 glycan view (Fig. 5B), the epitope of a glycan-dependent Nab utilizing N276 and a V5 loop N-terminal (N460) or a V5 loop central PNGS (N463) might include N49 (C1). As seen from a C3 glycan view (Fig. 5C), the epitopes of Nabs utilizing a V5 loop C-terminal (N465) or V5 loop central PNGS (N463) might include C2 (N295), C3 (N334 and N339), or V4 PNGS (N386, N392, and N397). N463 occupies a central position in the V5 loop, suggesting it might be available to form glycan epitopes with both C1 and C3 PNGS. The C1 glycan view (Fig. 5B) also shows that N49, N88, and N234 encircle N97, the C1 PNGS introduced to the EN Clade AE viral population (Fig. 2A).

## DISCUSSION

*Env* evolves in response to immune selection within an individual and serum breadth (neutralization of heterologous viruses) can occur concomitantly with viral escape (52, 53). The glycans that populate the surface of Env offer protection from the immune system (7, 54, 55) but are also the targets of neutralizing antibodies (2, 17) and therefore vulnerable to selective loss. In our study of viruses recovered from two elite neutralizers and five individuals showing viral control, the absence of select PNGS corresponded to significantly lower median IC_50_ values of bN-mAbs targeting V2, V3/C3, and CD4bs epitopes, but their absence alone was not predictive of serum breadth. Whereas both the EN and controller virus populations showed evidence of selection driven by glycan-dependent antibodies, the EN viruses exhibited positive selection at PNGS shielding immunogenic bNAb epitopes, as well as the introduction of glycan to the C1 domain. Positive selection at shielding PNGS suggested constraints on the escape pathways of glycan-dependent Nabs. An EN-derived antigen exhibiting a rare C1 PNGS showed a strain-specific increase in cross-reactive immune responses overlapping a VRC01 epitope contact.

EN Clade AE had an N334-based viral population that showed evidence of having elicited a VRC01-like bNAb (40) through mutations occurring at known epitope contacts in the C1, loop D, and β23/V5 domains (44–46, 56). Although this type of within-epitope polymorphism might be a component of bNAb maturation (5, 9, 57), polymorphism of PNGS in the C3 (N334), V4 (N392, N398), and V5 (N460, N465) strongly suggested a role for co-occurring immune selection by glycan-dependent antibodies. N465 and N392 are proximate to the VRC01 epitope, and on average, the absence of either glycan rendered viruses more sensitive to VRC01. A PNGS positioned at HXB2 465 would directly shield a VRC01 contact region in the V5 domain (44) that was central to the elicitation of an immune response to β24/C5 consensus peptides. Although an ^465^SEI^467^ residue combination, as seen in EN Clade B viruses, was associated with an abrogated local response, all EN Clade AE viruses retained T467 with positive selection of the residue at 465. The discovery of a C3/N465 epitope that blocked a V5-directed immune response elicited by BG505 (clade A) immunization (47) supported our prediction of an epitope utilizing C3 and V5 glycans (N334-N392-N465) in EN Clade AE. A polymorphic N234-N398-V5 N-terminal glycan combination, as seen in both the EN Clade AE and the clade C CH505 viral populations, was also suggestive of a glycan epitope. The lack of either a longitudinal or proviral sample from EN Clade AE limited our understanding of early viral evolution accompanying the putative development of a VRC01-like antibody. However, positive selection at N465 plus data illustrating the cross-reactivity of the β24/C5 region supported the concept that the loss of a glycan for escape from a glycan-dependent Nab conflicted with maintaining shielding of an immunogenic portion of the VRC01 epitope in an EN viral population.

Viruses recovered from the donor 45 individual from which VRC01 was first isolated (48) also exhibited positive selection at residues within the bNAb epitope (including the central residue of the N276 sequon), and at residues affecting formation of N49, N335, and N444. Here, the introduction of N339 formed a 2G12-like plus N463 or C3-V4-V5 loop central glycan combination that alternated with N335-N406-N444. The introductions of N49 (C1) and N276 (loop D) formed another putative glycan epitope that reflected a polymorphic combination seen in VC5 viruses (C1-loop D-V5). As evidenced by positive selection at N49 and residue 277, the escape pathway of an N276 glycan-dependent antibody in donor 45 may have been constrained by the VRC01 bNAb response where the loss of N276 would have increased viral sensitivity to VRC01 (46).

The pathway to serum breadth in EN Clade B appeared polyclonal in nature with the positive selection detected at the central residue of the N332 sequon and at a CD4bs contact suggestive of antibodies to both epitopes. Additional positive selection pointed to a co-occurring 2G12-like antibody that would have overlapped the N332 bNAb epitope (28). As the removal of either N295 or N392 could abrogate both binding and neutralization of 2G12 (20), it is surprising for viral escape from a 2G12-like antibody to exhibit positive selection at its epitope unless loss of those PNGS were increasing viral sensitivity to neutralization by an alternate specificity. Similarly, viral escape from an N332-dependent bNAb such as PGT121 could easily have occurred by the loss of N332 (20). But, as documented when N332 bN-mAbs were administered alone (*in vivo*) (58) but not in combination with CD4bs bN-mAbs (59), N332 was maintained in the EN Clade B viral population. However, the more immunogenic ^332^NLS^334^ PNGS sequon present in the basal virus evolved to the less immunogenic ^332^NIT^334^. Interestingly, the positive selection detected at the central residue of the 276 PNGS that shielded loop D in donor 45 viruses led to the appearance of a similarly coded glycosylation sequon (^276^NLS^278^) in a later sampling year. The cross-reactive responses elicited by an EN Clade B construct with ^332^NLS^334^ suggest that ^276^NLS^278^ might also be an important feature of a vaccine antigen.

As lower median neutralization titers for PGT121 (N332 epitope) and VRC01 (CD4bs epitope) were found to be associated with viruses lacking either N295 or N392, immune responses to both epitopes in concert with a 2G12-like Nab could conceivably have driven the type of viral diversification that associates with bNAb development (9, 11, 14, 46, 60). This scenario agrees with the observation that broad neutralizing activity often associates with the presence of 2G12-like antibodies (61) and that more complete viral escape within individuals exhibiting cross-reactive serum responses coincides with increases in variable loop lengths and their PNGS counts (62, 63). Consistent with these generalities, while neutralization escape for the majority of EN Clade B plasma viruses largely corresponded to the loss of N295 and N339 but retention of N392, it also occurred in concert with expansion and increased glycosylation of the V2 loop (34). Both the donor 45 and VC1 viral populations exhibited large increases in V1 loop lengths that associated with reductions in VRC01 sensitivity.

The possibility that HIV-1 might be evolving to minimize the formation of glycan epitopes is supported by the observation that the frequencies of 2G12-like glycan-dependent epitopes differ between clades. As escape from a glycan-dependent Nab could result in a breach of the glycan shield that allows development of a neutralizing response against HIV-1 (47, 64–66), there would likely be strong selective pressure favoring mutation of underlying immunogenic residues for viral escape instead of PNGS introductions that form glycan epitopes. The higher frequency of I467 versus a PNGS at 465 in clade B and clade C viruses could show dominance of an escape pathway that would avoid formation of an N465-dependent epitope. Similarly, the multi-individual correlation found for greater VRC01 sensitivity in the absence of N295 may simply reflect higher frequencies of glycan combinations lacking N295 together with more VRC01 epitope-adjacent glycans such as N392 and/or N465. However, a PNGS introduction that did not form a glycan epitope could effectively reduce neutralization sensitivity. Whereas selection by a glycan antibody describes the absence of N465 from a subset of EN Clade AE plasma viruses as well as the VC1 proviruses, the re-introduction of N465 to the VC1 plasma viruses following the loss of N295 and N339 (in concert with lengthening of the V1 loop) corresponded to serum neutralization resistance. Thus, the inadvertent creation of an N465 glycan epitope for shielding of immunogenic VRC01 contacts could have created a conflict for escape from a glycan antibody in EN Clade AE.

A third outcome of glycan introduction might be an increase in cross-reactivity. As shown by the C1 response elicited by the AN1 antigen, the sequential introduction of N49 and N234 to the EN Clade B viral population was associated with a heightened regional immune response overlapping a VRC01 epitope contact, possibly by defining a C1 glycan hole rather than filling it. Remarkably, similar glycan introductions were seen for the donor 45 viral population that produced VRC01. The role of N444, which was also introduced to and positively selected in the donor 45 and EN Clade B viral populations, is unclear from this study but could conceivably have affected cross-reactivity of CD4bs antibodies. N444 is a rarely observed glycan (<7% conserved [67]) whose introduction would encompass a cys378-cys445 disulfide bond. This bond creates a symmetrical gp120 structure between the C3/CD4 binding loop and β23/V5 domains displaying PNGS at 356, 362, 460, and 463 (68).

A limitation of our study is the inference that glycan-dependent antibodies were elicited for the polymorphic glycan combinations observed in each participant. A decline in autologous serum neutralization titers for the loss of a putative glycan epitope was only implied for the VC2 viral population. However, the transience of viral glycan combinations comprising putative 2G12-like/V5 epitopes in multiple individuals is supportive of the concept that glycan loss for viral escape from glycan antibodies underlies shifting glycan landscapes (7). Therefore, the persistence of PNGS that were components of glycan-dependent epitopes in the two EN viral populations with divergent infections supports the argument that the escape pathways of glycan antibodies were constrained by the co-occurring bNAb response. Ideally, longitudinal sequence data from additional EN individuals would be necessary to determine whether this finding is a general feature of natural viral evolution accompanying development of the EN phenotype.

This study found that introductions of rarely observed PNGS to the C1 domain and positive selection of glycans shielding immunogenic bNAb epitope positions characterized viral evolution in two ENs with divergent infections. However, these parallelisms were not necessarily mutually exclusive. For instance, positive selection detected within bNAb epitopes might stem from overlapping B-cell lineages (12). Nonetheless, elements of the EN pattern of glycan evolution were also seen in the donor 45 viral population which has ramifications for eliciting VRC01-like responses for a vaccine. Current immunization strategies that employ deletion of conserved PNGS proximate to the CD4bs, including N197 and N276 (respectively 97% and 95% conserved across clades), can induce extremely high neutralization titers that neutralize autologous virus (65). Priming with a deglycosylated BG505 immunogen that was engineered to engage the VRC01 germline and then boosting with the glycosylated trimer generated VRC01-like bNAb precursors that also neutralized autologous virus (69, 70). However, the failure to neutralize heterologous viruses points to the effectiveness of wild-type glycosylation in blocking neutralizing responses directed to rare glycan holes (43).

This raises the question of whether rarely observed glycan deletions (N276, N197) are necessary for elicitation of VRC01 precursors (71). Even though the donor 45 phylogeny suggested that N276 absence preceded VRC01 evolution, the observation that serum breadth can develop after repeated rounds of viral escape would be consistent with the later appearance of an ^276^NLS^278^ sequon for inducing cross-reactive loop D responses, and supports retention of N276 in a vaccine priming immunogen (72). In addition, N197 was present in all sampled donor 45 viruses, and its singular absence from the VC1 virus population (109920_025 with an autologous serum neutralization titer of ID_50_ 1/dilution > 4,000) suggests high intrinsic serum neutralization titers, while perhaps underlying viral control, might not be a critical component of breadth. Whereas high autologous serum neutralization titers also characterized the proviruses recovered from VC3, they were not a feature of VC4 viruses whose sera exhibited a level of serum breadth second only to the ENs. However, there was a remarkable concurrence between immune responses stimulated by a VC4 plasma virus construct lacking N88 (98% conserved), N295, and N392 with those elicited by the separate EN Clade B antigens. The VC4 antigen elicited high median cross-reactive responses to both the C1 and V3/C3 epitopes, with only an EN Clade B antigen eliciting a significantly greater C1 response.

The C1 domain of HIV-1 represents a relatively glycan-free region in most wild-type viruses. Based on the comparison of the C1 peptide responses for the two EN Clade B antigens, a donor 45 construct exhibiting a rare N49/N234 glycan combination might also promote cross-clade recognition of a VRC01 contact that would not be blocked by ordinary wild-type glycosylation. Thus, a uniquely glycosylated donor 45 virus that emerged in a later sampling year that was not VRC01-sensitive (i.e., 45_06A1) might be a logical starting point for a backbone vaccine antigen. Initial modifications to such a backbone would entail restoring variable loop lengths to the sensitive provirus level and introducing cross-reactive C1 and loop D residues. Thereafter, as seen in the donor 45 phylogeny, shifts (rather than deletions) of V5 PNGS and variation of a C4 domain PNGS (N444) might be required to expand the breadth of VRC01 pre-cursors to produce a protective vaccine.

## MATERIALS AND METHODS

### Clinical specimens and collection of env sequences

Plasma and peripheral blood mononuclear cell (PBMC) samples for three subjects, VC1, VC2 (aka EN2 [36]), and VC3 (aka EN3 [36]) were kindly supplied by Steven Deeks via the SCOPE study cohort (University of California San Francisco, San Francisco, CA) (73) and screened for neutralization breadth as previously described for VC3 (aka EN3 [36]) and EN Clade B (aka EN1 [34]), including a panel of five viruses predictive of the elite neutralizer (EN) phenotype (1). All SCOPE participants provided written informed consent, including permission to store and use specimens for research. The University of California, San Francisco Institutional Review Board (IRB) approved consent forms and protocols for the Northern California location. Viral loads of <10,000 RNA copies/mL after >10 years following infection are indicative of viral control (74). At the time of sampling, VC1 had been infected by HIV-1 for 22 years. Clinical records for VC1 in the 11 years prior to sampling indicated the subject had maintained a viral load of <150 copies/mL for the first seven years and between 200 and 2,500 copies/mL for 4 years thereafter. Similarly, at the time of sampling, VC2 had been infected for 15 years with a viral load of <700 copies/mL. Plasma and PBMC samples for VC4 and VC5 were obtained from the Women’s Interagency HIV Study (WIHS, Baltimore, MD) (75–77). VC4 had maintained viral control for 17.5 years at <500 copies/mL. The date of infection for VC5 was unknown, but this subject had an estimated viral load of <6,500 cells/mL at the time of sampling and was not on antiviral therapy. All WIHS participants provided written informed consent, including permission to store and use specimens for research under IRB guidelines.

For these studies, functional virus envelopes were randomly selected for sequencing from plasma virus and cell-associated virus (provirus) using a phenotype infectivity method developed by Monogram Biosciences (South San Francisco, CA) (6). All full-length, functional *env* clones were recovered from plasma virus RNA using the PhenoSense Entry assay system, and from proviral DNA in PBMCs using the Trofile DNA assay system as previously described (34). Monogram Biosciences performed all sequencing of *env* and tested the expressed pseudoviruses for sensitivity to neutralization by autologous polyclonal serum as well as a panel of broadly neutralizing antibodies provided by either the NIH AIDS Reagent Program (PGT121, PGT145, 35022), Polymun Scientific (Vienna, Austria) (PG16, 4E10) or produced in-house (PG9, PGT128, VRC01).

Also in accordance with IRB guidelines, plasma from EN Clade AE, previously described as subject T500107 (35), was kindly supplied by Ruengpung Sutthent and Navin Horthongkham (National HIV Repository and Bioinformatics Center, Department of Microbiology, Faculty of Medicine, Siriraj Hospital, Mahidol University, Bangkok, Thailand). The EN Clade AE subject had been identified through screening of rejected blood donations by TZM-bl neutralization assay (78), and its plasma was found to potently neutralize a broad panel of HIV-1 isolates (35). EN Clade AE was not on antiviral therapy and had been HIV positive for an indeterminate period. Recovery of *env* from plasma and viral sequencing was also performed by Monogram Biosciences.

### Phylogenetic analyses

The plasma virus and provirus gp160 sequences were aligned using the MUSCLE algorithm in Geneious v 5.6.7 (79). Maximum likelihood (ML) gene trees were constructed using the General Time Reversible (GTR) model, with gamma distributed rate heterogeneity (six discrete categories) and a proportion of invariant sites (G + I) in MEGA6 (80) using the clade B JRCSF isolate as an outgroup for the Clade B viruses and 92TH021 for the EN Clade AE viruses. Measures of nucleotide diversity (H_d_, θ_Π_, and θ_S_), absolute divergence (d_XY_), and Tajima’s D values were obtained using DnaSP v5.10 (81). Frequencies of amino acids were calculated in Geneious from the 2013 HIV-1 sequence database (4,907 sequences) of the Los Alamos National Laboratory (LANL) or referenced from other publications.

### Tests for positive selection

The codeml program of the PAML v4.7 package (39) was used to test for the presence of positively selected sites (i.e., codons) in the gp160 gene. Using the ML trees described above, likelihood ratio tests were performed comparing the scores obtained from models M7 and M8. Model M7 (beta) assumes that ω ratios follow a beta distribution constrained in the interval (0, 1). Under model M8 (beta & ω), an additional class of sites is added to the M7 model that allow for a proportion to have *d_N_*/*d_S_* ratios (i.e., ω ratios) estimated from the data to exceed unity. To evaluate the presence of local optima on the likelihood surface (cf. 82), model M8 was re-run with starting ω values of 0.5, 1.0, 1.5, and 3.0. If sites with ω ratios > 1 were identified, the Bayes empirical Bayes method of Yang et al. (83) was used to estimate posterior probabilities for each codon.

### Statistical analyses

Predicted N-linked glycosylation sites (PNGS) were determined by manual inspection in all viruses. After identifying the PNGS or residues of interest that occurred at the same position in all viruses, non-parametric Mann-Whitney U tests (GraphPad PRISM 6, GraphPad Software Inc., La Jolla, CA) were used to compare the median bN-mAb neutralization titers for their presence or absence.

### Structural mapping of mutations

Locations of mannose patch PNGS and amino acids were mapped onto the structure depicted by PDB 4TVP (51) using PyMOL (PyMOL Molecular Graphics System, v0.99; DeLano Scientific, Palo Alto, CA).

### Immunizations and peptide analyses

Rabbit immunization studies were conducted in accordance with the Animal Welfare Act at Pocono Rabbit Farm & Laboratory (Canadensis, PA), a facility accredited by Association for Assessment and Accreditation of Laboratory Animal Care International (AAALAC 925) and assured by the National Institute of Health (NIH) and Office of Laboratory Animal Welfare (OLAW A3886-01). New Zealand White rabbits were immunized with three 200 µg/doses of rgp120 produced in HEK293S GnTI^-^ cells (ATCC CRL-3022). The results presented in this paper include data from two rabbits each for the EN Clade B, MN, and A244 antigens. Data from five rabbits are presented for VC1_005 and VC4_226 and from four rabbits for VC3_071. Primary immunizations were formulated with Complete Freund’s Adjuvant (CFA) while subsequent boosts were formulated with Incomplete Freund’s Adjuvant (IFA). Serum immune responses were measured for final bleeds (day 147) utilizing a linear epitope mapping assay conducted by Xiaoying Shen and Georgia Tomaras at Duke University (Durham, NC) (84). Data from each rabbit are presented as a sum of the signals of three overlapping peptides covering indicated sequence for clades A, B, C, D, and AE.

## Data Availability

The sequences described in this publication have been deposited in GenBank with accession numbers for nucleotide and amino acid sequences as follows: EN Clade B (aka EN1): MK164661-MK164683; VC3 (aka EN3): MN516428-MN516441; EN Clade AE (aka T500107): PV769268-PV769298; VC1: PV769299-PV769318; VC2: PV769319-PV769338; VC4: PV769339-PV769358; and VC5: PV769359-PV769378. Supplemental material may be found at https://doi.org/10.6084/m9.figshare.29534318.v1.
